# Chronic unilateral groin pain in a young patient who injects drugs: a case report of needle fragment retentions

**DOI:** 10.1186/s13722-022-00309-2

**Published:** 2022-05-13

**Authors:** Heidi Laukkala, Otso Arponen, Mika O Murto, Olli PO Nevalainen

**Affiliations:** 1 Hatanpää Health Center, Tampere, Finland; 2 Department of Radiology, Tampere University Hospital, Tampere, Finland; 3 Faculty of Medicine and Health Technology, Tampere University, Tampere, Finland; 4 Department of Surgery, Tampere University Hospital, Tampere, Finland

**Keywords:** People who inject drugs, Needle, PWID, Pain, Asymptomatic

## Abstract

**Background:**

Subcutaneously retained needle fragments in people who inject drugs (PWIDs) are a possible cause of local symptoms, most commonly pain and infections. It remains unknown how common retained needle fragments are among PWIDs.

**Case presentation:**

A young PWID consulted a primary care physician due to chronic left-sided groin pain. The patient suspected retention of a broken needle as the cause. She had used a re-used needle 3 months earlier. A plain pelvic radiograph confirmed a needle fragment in the patient's left groin, and a computed tomography scan located it adjacent to the femoral artery and vein. Another asymptomatic needle fragment was found in the right groin.

**Conclusion:**

Needle fragments are possible causes of local symptoms among PWIDs. The clinical examination presents a potential risk of needlestick injury to the examiner, especially because patients may not be aware of all needle fragments as some are asymptomatic.

## Background

The prevalence of needle fragment retention in people who inject drugs (PWIDs) is unknown as there are no studies of representative populations described in the literature. In 2002, Norfolk and Gray stated that 14 of 70 (20%) PWIDs held in Bristol police custody reported a total of 23 broken needles during their injecting history and noted that the majority (61%) of the broken needles had been re-used [1].

Subcutaneous needle retention in PWIDs commonly causes local symptoms such as pain and infections. Additionally, previous case reports have described serious complications from broken needles, including needle embolisms in the brain, heart, or lungs [2–6]. Nonetheless, the benefit of removing a needle fragment that causes local symptoms must be balanced against possible harms (such as operation-related risks, possible complications, and the risk posed to the surgical staff if the patient is infected with human immunodeficiency virus or viral hepatitis). As far as we know, there are no reports on simultaneous symptomatic and asymptomatic bilateral needle fragments in the same patient.

## Case presentation

A young female with a history of polysubstance use and an untreated hepatitis C infection visited a primary care physician due to left groin pain. The patient herself suggested that the pain could be caused by a broken needle that had remained embedded underneath the skin. She recalled that around 3 months before the consultation, she was desperate to find a suitable vein for drug injection. During the consultation, she demonstrated with a violent hand movement how she virtually stabbed herself in the groin with the needle.

**Fig. 1:**
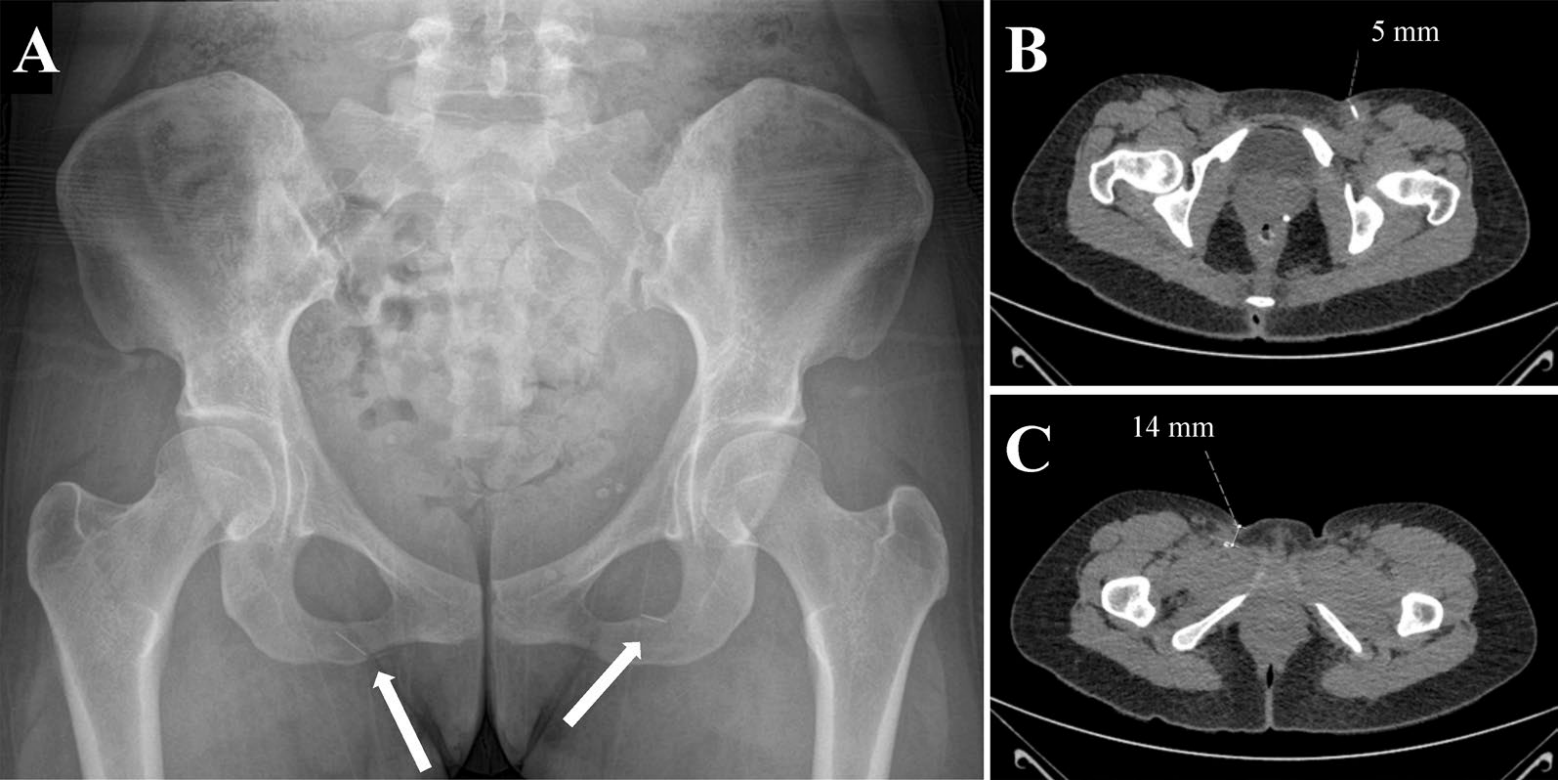
A young female patient who injects drugs with two broken needle fragments roughly 10 mm long. The fragments were visible on a plain radiograph (**A**). The fragments could not be visualized in the ultrasound assessment. The patient underwent subsequent computed tomography imaging to precisely locate the fragments. The fragment on the left side was close (5 mm) to the skin and adjacent to the femoral artery and veins (**B**). The tip of the fragment on the right side was located 14 mm from the skin; however, it did not pose a threat to either arterial or venous structures

Because of a known hepatitis C infection and the possibility of a needle fragment, the physician did not use gloved fingers to palpate the area but rather employed forceps to identify possible metal beneath the skin. The skin in the groin area had tiny scars from frequent drug injections. Otherwise, inspection and clinical examination revealed nothing remarkable. A pelvic radiograph was requested and revealed needle fragments in the left and right sides of her groin (Fig. 1A). The patient was unaware of the asymptomatic needle in the right groin. A radiologist attempted to identify the exact location of the needle fragments using ultrasound, but these could not be visualized. The patient was referred to a tertiary care hospital for surgical assessment. The surgeon prescribed a computed tomography scan (Fig. 1B and C) after verifying that the patient's human chorionic gonadotropin level was normal to rule out possible pregnancy. She was then referred to surgery for the removal of the needle fragments. The patient did not come to the surgical clinic at the appointed time, likely reflecting her unstable living situation with continuing drug use; this could also explain why she consulted a physician for the first time only months after the needle fragmentation incident. Attempts are being made to engage the patient in substance use disorder treatment. Furthermore, the patient has been made aware of the city's and several non-profit organizations' free syringe services programs for PWIDs.

## Discussion and conclusions

Repeated injection of drugs damages veins, leading to vein sclerosis [7]. Consequently, due to damaged and inaccessible peripheral veins, some PWIDs turn to central veins such as those of the groin and neck, and they sometimes even inject subcutaneous or muscle tissues [7]. Indeed, patients with needle retentions near central veins are likely to have a prolonged drug use history. When a PWID presents with local pain (or inflammation) in an injection area, clinicians should ask about the possibility of fragmented needles. To avoid a needlestick injury, clinicians should be aware that retained needles can be asymptomatic. In addition, a needle fragment can be a subtle incidental finding in imaging examinations and thus can be easily overlooked if the examiner is unaware of the patient's background. It is therefore important that the individual's intravenous drug use is disclosed when referring the patient for radiological examinations. Depending on the orientation and depth of the needle in the tissue, needle fragments may not always be identified using ultrasound, as this case demonstrated. 

We did not ask the patient why she sought medical attention only 3 months after the suspected fragmentation of a needle. In general, PWIDs may have severely delayed presentation to health care for medical problems because of previous negative experiences with stigma and withdrawal. Furthermore, PWIDs are at a higher risk of treatment failures [7]. Although Finland has several low-threshold outpatient health services for PWIDs, including health centers that the patient can contact for immediate on-call consultation, some PWIDs may choose not to contact health care providers. In our municipality, opioid use disorder treatment involves both administration of medication as well as organization of social support, contact with the unemployment office, and a clinical follow-up. After recovery has started, patients are referred to the local primary care health center for follow-ups. At present, there are no supervised injection facilities in use in Finland. Supervised injection facilities could constitute places to reach marginalized and homeless PWIDs, provide information on outpatient services, teach safer injection techniques, and exchange needles. There is growing evidence that these facilities reduce injection complications [8, 9]. Furthermore, medical staff could teach or help to organize information sessions on performing injections safely for PWIDs to reduce injection complications.

Once a fragmented needle is detected, the benefit of its removal must be balanced against the risks (including the risk posed to the operator). We empirically suggest that, in addition to managing the fragmented needle, clinicians should educate PWIDs of the services available to them as well as encourage PWIDs to use new needles and to seek medical help when needed.

## Data Availability

All relevant data is presented within the manuscript.

## Abbreviation

PWID: Person who injects drugs
PWIDs: People who inject drugs
